# The novel functional nucleic acid iRed effectively regulates target genes following cytoplasmic delivery by faint electric treatment

**DOI:** 10.1080/14686996.2016.1221726

**Published:** 2016-09-16

**Authors:** Mahadi Hasan, Noriko Tarashima, Koki Fujikawa, Takashi Ohgita, Susumu Hama, Tamotsu Tanaka, Hiroyuki Saito, Noriaki Minakawa, Kentaro Kogure

**Affiliations:** ^a^ Department of Biophysical Chemistry, Kyoto Pharmaceutical University, Kyoto, Japan; ^b^ Tokushima University Graduate School of Biomedical Sciences, Tokushima, Japan

**Keywords:** iRed, 4′-thioDNA, RNAi effect, faint electric treatment, cytoplasmic delivery, 30 Bio-inspired and biomedical materials, 211 Scaffold / Tissue engineering / Drug delivery, 600 Others: Nucleic acids

## Abstract

An intelligent shRNA expression device (iRed) contains the minimum essential components needed for shRNA production in cells, and could be a novel tool to regulate target genes. However, general delivery carriers consisting of cationic polymers/lipids could impede function of a newly generated shRNA via electrostatic interaction in the cytoplasm. Recently, we found that faint electric treatment (fET) of cells enhanced delivery of siRNA and functional nucleic acids into the cytoplasm in the absence of delivery carriers. Here, we examined fET of cells stably expressing luciferase in the presence of iRed encoding anti-luciferase shRNA. Transfection of lipofectamine 2000 (LFN)/iRed lipoplexes showed an RNAi effect, but fET-mediated iRed transfection did not, likely because of the endosomal localization of iRed after delivery. However, fET in the presence of lysosomotropic agent chloroquine significantly improved the RNAi effect of iRed/fET to levels that were higher than those for the LFN/iRed lipoplexes. Furthermore, the amount of lipid droplets in adipocytes significantly decreased following fET with iRed against resistin in the presence of chloroquine. Thus, iRed could be a useful tool to regulate target genes following fET-mediated cytoplasmic delivery with endosomal escape devices.

## Graphical abstract

**Figure:**
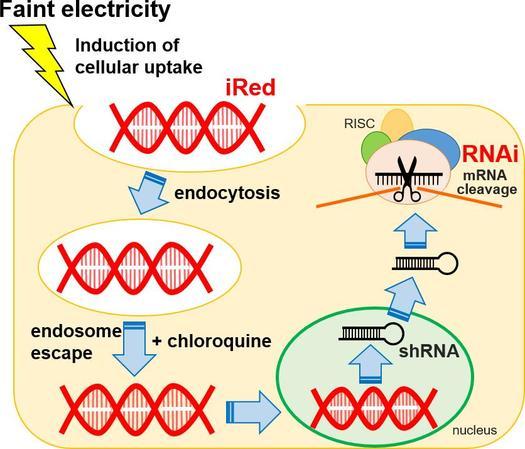

## 1. Introduction

**Figure 1.**
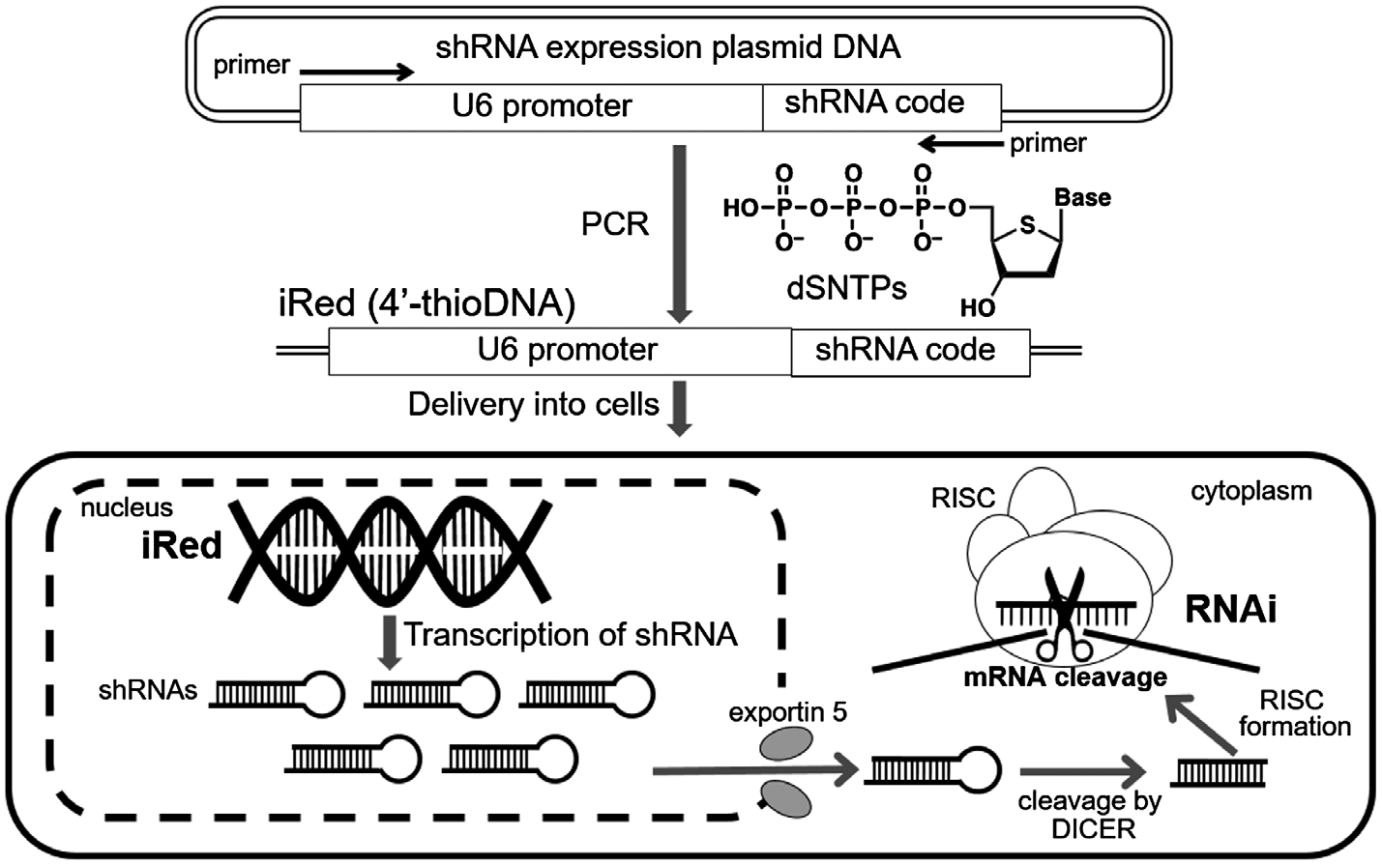
Schematic image of dSC iRed preparation and transcription of shRNA for RNAi effect.

RNA interference (RNAi) is a promising method to regulate the expression of target genes, including those that are implicated in various diseases. Although small interfering RNA (siRNA) and short hairpin RNA (shRNA) produced from plasmid DNA are often used to induce the RNAi effect, each tool has some drawbacks. For instance, in the absence of chemical modification, the stability of siRNA is lower than that of DNA, and non-specific RNAi can occur.[1,2] On the other hand, low delivery efficiency due to large molecular size and innate immune responses to CpG motifs present in shRNA-encoding plasmid DNA must be overcome.[3] The intelligent shRNA expression device (iRed) consisting of polymerase chain reaction (PCR)-amplified 4′-thioDNA was recently developed to address these issues (Figure 1).[4] iRed encodes a minimum sequence to give shRNA in cells, such as U6 promoter and shRNA-encoding region, in which any one type of adenine (A), guanine (G), cytosine (C), or thymine (T) nucleotide unit was substituted by each cognate 4′-thio derivatives, i.e. dSA iRed, dSG iRed, dSC iRed, and ST iRed respectively. As a result, potent RNAi effects were observed especially by introducing dSC iRed both *in vitro* culture cell system and *in vivo* tumor-bearing mouse model, without inducing innate immune response-dependent cytokine production. Thus, dSC iRed is an ideal molecule for nucleic acid-based medicines.

Step 1: dSC iRed was prepared by PCR amplification of the region encoding U6 promoter and shRNA in the presence of dSNTP; Step 2: delivery of dSC iRed into target cell nuclei; Step 3: shRNAs are transcribed from iRed in the nucleus. The shRNAs are exported from the nucleus; an RNAi effect is evident.

The approaches used to deliver nucleic acid-based molecules into cells are another important factor in the effective regulation of target genes. Although viral vectors or non-viral nanoparticles are often used to deliver RNAi devices, these approaches also have some drawbacks. Viral vectors present the risk of infection and immunogenicity, although those issues were recently minimized by various modifications, such as PEgylation.[5] Meanwhile, non-viral nanoparticles consist of cationic polymers/lipids, which can form complexes with negatively charged nucleic acids through electrostatic interactions.[6,7] Furthermore, general delivery carriers consisting of cationic polymers/lipids also reportedly participate in electrostatic interactions with mRNA or functional nucleic acids in target cells [8] such that a cationic non-viral delivery system could impede the function of shRNA transcribed from dSC iRed. Viral vectors and non-viral cationic nanoparticles may be unsuitable for dSC iRed delivery. Thus, as carriers induce undesired effects depending on carrier properties, other approaches without carriers are needed to promote effective cellular uptake of this shRNA expression device.

We recently succeeded in the transdermal delivery of functional nucleic acids, such as siRNA, using a physical technology that involves iontophoresis induced by faint electricity that promotes opening of intercellular junctions by activating cellular signaling.[9–11] Furthermore, we recently found that faint electric treatment (fET) enhanced cellular uptake and homogenous and rapid delivery of functional nucleic acids into cytoplasm by altering membrane potentials without promoting cytotoxicity.[12] Thus, fET would be a useful approach for the delivery of dSC iRed into target cells.

Here we examined the delivery of dSC iRed by fET, and the functionality of dSC iRed encoding luciferase shRNA to reduce luciferase expression in cells that stably express luciferase. To improve delivery efficiency, intracellular trafficking of extraneous macromolecules such as dextran labeled with fluorescein isothiocyanate (FITC-dextran) after fET was also evaluated. Furthermore, we assessed the effect of fET-mediated delivery of dSC iRed targeting resistin, a key adipokine in obesity, on lipid accumulation in 3T3-L1 adipocytes.

## 2. Materials and methods

### 2.1. Materials

Natural dNTPs were purchased from GE Healthcare Japan (Tokyo, Japan). 2′-Deoxy-4′-thiocytidine 5′-triphosphate (dSCTP) was prepared according to our previous reports.[13] Oligonucleotides were purchased from FASMAC (Kanagawa, Japan). Rhodamine-labeled anti-GFP siRNA (21-mer, 5′-gcugacccugaaguucauctt-3′, 5′-gaugaacuucagggucagctt-3′) was obtained from Invitrogen Life Technologies (Carlsbad, CA, USA). FITC-dextrans with average molecular weights (MW) of 10,000 and 70,000 were purchased from Tokyo Kasei (Tokyo, Japan) and Sigma-Aldrich, Inc. (St Louis, MO, USA), respectively. LysoTracker Red DND-99 was obtained from Thermo Fisher Scientific Inc. (Waltham, MA, USA). Chloroquine was purchased from Nacalai Tesque (Kyoto, Japan). Lipofectamine 2000 (LFN) was obtained from Invitrogen Life Technologies. Cell lysis buffer was purchased from Promega Corporation (Madison, WI, USA). The mouse melanoma cell line B16-F1 was obtained from Dainippon Sumitomo Pharma Biomedical Co., Ltd (Osaka, Japan), and stable transformants of B16-F1 cells expressing luciferase (B16-F1-Luc) were established in our laboratory.[14] These cells were cultivated in Dulbecco’s modified Eagle’s medium (DMEM) supplemented with 10% fetal bovine serum (FBS) at 37° C in 5% CO_2_. The 3T3-L1 cell line was purchased from the Japan Health Sciences Foundation (Tokyo, Japan). The culture and differentiation induction of 3T3-L1 cells were performed according to the procedure described below.

### 2.2. Preparation of dSC iRed

dSC iRed was prepared as previously reported.[4] For construction of shRNA expression plasmid DNA, oligonucleotides encoding shRNA and terminator sequences were ligated into *BamHI* and *EcoRI* sites in an shRNA expression cassette-containing the U6 promoter (RNAi-Ready pSIREN-RetroQ, Clontech, Mountain View, CA, USA) according to the manufacturer’s instructions. The inside sequences are luciferase (5′-aattcaaaaaacttacgctgagtacttcgacaaccaggagcactatcgaagtactcagcgtaag-3′) and resistin (5′-aattcaaaaaagcgctgctggtgccaaccctctcttgaagggttggcaccagcagcgcg-3′). To prepare dSC iRed, PCR was performed using the shRNA expression plasmid DNA as a template. The U6 promoter and shRNA sequences in the plasmid DNA were amplified in 20 μl KOD buffer containing KOD Dash DNA polymerase (0.05 unit μl^–1^, TOYOBO, Osaka, Japan), template plasmid DNA (0.1 fmol μl^–1^), 200 μmol l^–1^ dNTPs, and 0.5 μmol l^–1^ two primers (5′-primer; 5′- ttctctaggcgccggaattgaagatctgggc-3′ and 3′-primer; 5′-ccctacccggtagaattcaaaaaacttacgctg-3′ for luciferase target, or 5′-tcccctacccggtagaattca-3′ for resistin target). PCR was performed as follows: initial denaturation at 94° C for 15 s, 15 cycles of denaturation/amplification (94° C, 30 s; 62° C, 30 s; 72° C, 30 s), and final extension at 72° C for 15 min. Amplicons were subjected to 1% agarose gel electrophoresis, and the DNAs were purified with a High Pure PCR Product Purification Kit (Roche, Basel, Switzerland). After purification, a second PCR amplification using the purified DNA as a templates was performed. The 100 μl reaction mixture consisted of KOD Dash DNA polymerase (0.1 unit μl^–1^), template DNA (0.2 fmol μl^–1^), 200 μmol l^–1^ nucleoside 5′-triphosphate mixture containing dSCTP and three other types of dNTPs, and 1.25 μmol l^–1^ of primers. The second PCR was performed as follows: initial denaturation at 94° C for 15 s, 30 cycles of denaturation/amplification (94° C, 30 s; 62° C, 30 s; 72° C, 10 min), and final extension at 72° C for 15 min. The dSC iRed amplicons were purified with a High Pure PCR Product Purification Kit (Roche). FITC-labeled dSC iRed was prepared using this same protocol but with 5′-FITC labeled 5′-primer.

### 2.3. Faint electric treatment of cells

Cells were cultivated in 35 mm dishes for *in vitro* fET. The number of cells used is mentioned in each section below. After 18 h of cultivation, cells were washed with PBS, and then 800 μl serum free DMEM containing 100 pmol dSC iRed, or 100 pmol fluorescently labeled siRNA with or without 100 μM chloroquine was added to the cells. Ag-AgCl electrodes with a 2.5 cm^2^ surface area (3 M Health Care, Minneapolis, MN, USA) were placed into the dish, and cells were treated with a constant current of 0.34 mA cm^–2^ for 15 min.

### 2.4. Transfection and measurement of luciferase activity

B16-F1-Luc cells were cultivated at a density of 1 × 10^4^ cells in 35 mm culture dishes. After 18 h of incubation, cells were washed with PBS, and 1 ml serum free DMEM containing 100 pmol dSC iRed encoding anti-luciferase shRNA with or without 100 μM chloroquine was added before the cells were treated with electricity as described above. Three hours after fET, 1 ml DMEM containing 10% FBS was added, and the cells were further incubated for 21 h. After the incubation, the cells were lysed with Reporter Lysis Buffer (Promega) according to the manufacturer’s protocols. The luciferase assay substrate (Promega) was added to cell lysates, and chemiluminescence intensity was measured by a luminometer (Luminescensor-PSN, ATTO Corp., Tokyo, Japan). The total protein concentration was measured with BCA protein assay kit (Thermo Scientific).

### 2.5. Measurement of fluorescently labeled siRNA uptake after electric treatment

To measure the amount of siRNA cellular uptake, B16-F1 cells were seeded at a density of 1 × 10^5^ cells in 35 mm culture dishes. After 18 h of cultivation, cells were washed with PBS and 1 ml serum free DMEM containing 100 pmol rhodamine-labeled siRNA was added into the dish. fET was then performed as described above. After the fET, the cells were incubated for 3 h at 37° C. The cells were then lysed with reporter lysis buffer (Promega) according to the manufacturer’s protocols. The fluorescence intensity of the cell lysates was measured with an Infinite 200 microplate reader (Tecan Group Ltd, Männedorf, Switzerland) at excitation and emission wavelengths of 546 nm and 576 nm, respectively.

### 2.6. Confocal laser scanning microscopy of cells after electric treatment

For evaluation of intracellular delivery of FITC-dextran10,000 or FITC-dextran70,000, 5 × 10^4^ cells were seeded on 0.002% poly-L-lysine coated 35 mm glass bottom dishes. After 18 h, cells were washed with PBS and treated with fET (0.34 mA cm^–2^ for 15 min) in the presence of 5 μM FITC-dextran10,000 or 0.5 μM FITC-dextran70,000. After fET, the cells were incubated for 30 min at 37° C in 5% CO_2_. Then, 0.1 μl LysoTracker Red DND-99 was added to the culture medium, and the cells were incubated for 30 min at 37° C. After incubation, the cells were observed with a LSM 700 confocal laser scanning microscope (Carl Zeiss, Oberkochen, Germany).

#### 2.7. 3T3-L1 cell culture and induction of differentiation

3T3-L1 pre-adipocytes were cultured and differentiation into adipocytes was induced as previously reported.[15] The cells were cultured in basal medium (DMEM supplemented with 10% FBS, 10 mM HEPES, 0.2% NaHCO_3_, 4 mM L-glutamine, 3.5% (w/v) glucose, 0.2 mM ascorbate, 1 mM T3 and 30 μM T4) at 37° C under a humidified atmosphere containing 5% CO_2_. The day on which cells reached confluence was defined as day 0. Adipocyte differentiation was induced by incubation in differentiation medium (basal medium containing 500 μM 3-isobutyl-1-methylxanthine, 1 mM dexamethasone and 1.6 μM insulin) from days 0 to 2, followed by induction of maturation in maturation medium (basal medium containing 1.6 μM insulin and 15 μM D-biotin). On day 2 cells were treated with fET in the presence of dSC iRed encoding anti-resistin shRNA as described above.

### 2.8. Oil Red O staining of lipid droplet in adipocytes

The 3T3-L1 cells were fixed with 10% (v/v) formalin solution (Wako Pure Chemical Industries, Osaka, Japan) two days after fET and the change to maturation medium. The fixed cells were incubated with 18 mg/ml Oil Red O in a 60% (v/v) 2-propanol solution for 20 min at room temperature and the lipid droplets in cells were observed by microscopy. Then, Oil Red O dissolved in the lipid droplets was extracted with 100% 2-propanol, and its relative concentration was determined by measuring absorbance at 540 nm.

### 2.9. Confocal laser scanning microscopy of intracellular trafficking of FITC-labeled dSC iRed in 3T3-L1 cells

For evaluation of intracellular fate of the dSC iRed following the fET-mediated transfection, 3T3-L1 cells were seeded on 0.002% poly-L-lysine coated glass bottom dishes at the density of 0.5 × 10^5^ cells/dish. After 18 h of cultivation, cells were washed with PBS and 1 ml serum free DMEM medium containing 100 pmol FITC-labeled dSC iRed with or without 100 μM chloroquine (Wako Pure Chemical Industries) was added and treated with fET (0.34 mA cm^–2^ for 15 min). After fET, cells were incubated for 2.5 h at 37° C and 5% CO_2._ Then, Lyso tracker Red DND-99 (Thermo Fisher Scientific) (final concentration 250 nM) was added to stain endosomes/lysosomes and cells were incubated for 30 min. After incubation, Hoechst 33342 (Sigma-Aldrich) was added to the culture medium followed by 5 min incubation for staining of nucleus. After washing with PBS(-), cells were observed with confocal laser scanning microscope A1R+ (Nikon Co. Ltd, Tokyo, Japan).

### 2.10. Statistical analysis

Statistical analysis was performed using one-way ANOVA followed by Turkey–Kramer honest significant difference (HSD) test. *P*-values < 0.05 were considered to be significant; they were evaluated using JMP software (SAS Institute Inc., Cary, NC, USA).

## 3. Results and discussion

### 3.1. Transfection of dSC iRed via fET

**Figure 2.**
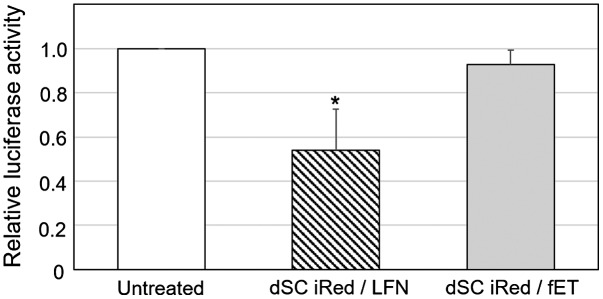
Transfection of dSC iRed encoding anti-luciferase shRNA with fET into cells stably expressing luciferase.

Cells stably expressing luciferase were first transfected with either fET with dSC iRed encoding anti-luciferase shRNA or dSC iRed/LFN lipoplexes, and the transfection efficiency was compared. Although the knockdown effect was not so potent, the luciferase activity was reduced by dSC iRed/LFN lipoplexes (Figure 2), indicating that shRNA produced from dSC iRed in the cells had functionality as an RNAi device as reported previously.[4] Previously, we reported the RNAi effects of typical luciferase-siRNA by fET and lipofectamine 2000(LFN)/luciferase-siRNA lipoplex on luciferase activity of B16-F1 cells.[12] The silencing effect of dSC iRed/LFN lipoplex was almost the same as that of LFN/luciferase-siRNA lipoplex. Although LFN/luciferase-siRNA lipoplex is known as the potent siRNA vector, the silencing effect was not so potent. The reason for this would be due to the very high luciferase activity of this B16-F1 clone cells. Thus, although activity of LFN/luciferase-siRNA lipoplex was potent, it was suggested that silencing effect was relatively low on this cell line. In contrast, dSC iRed/fET showed no significant effect on luciferase activity (Figure 2).

Luciferase activity of cells expressing luciferase was measured 24 h after fET (0.34 mA cm^–2^, for 15 min) in the presence of dSC iRed solution. Lipofectamine 2000/ dSC iRed lipoplex was used as a positive control. LFN: lipofectamine 2000, fET: faint electric treatment. Data were obtained by three separate experiments, and are expressed as mean ± SD. *p<0.05 versus untreated.

### 3.2. Cellular uptake of functional nucleic acids by fET

**Figure 3.**
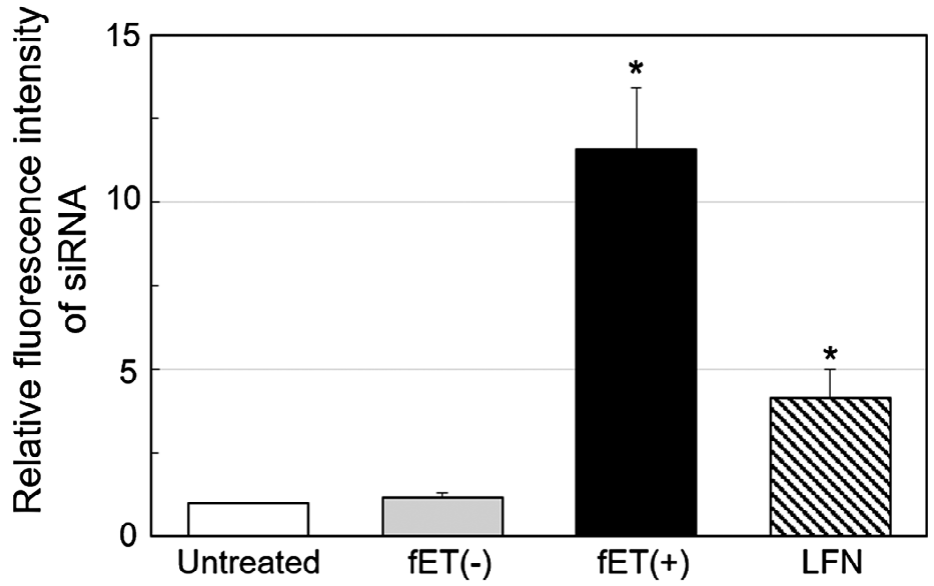
Relative fluorescence intensity of cells after fET with fluorescently labeled siRNA.

To determine why dSC iRed/fET transfection produced no significant RNAi effect, cellular uptake of functional nucleic acids following fET was examined. To perform an experiment briefly, fluorescently labeled anti-GFP siRNA was used instead of dSC iRed. The fluorescence intensity of cells treated with LFN/siRNA lipoplexes increased, indicating that siRNA could indeed be taken up by the cells. Surprisingly, the fluorescence intensity of cells treated with electricity was significantly higher (threefold) than that for the lipoplexes (Figure 3). This result suggests that cellular uptake induced by fET is more effective than cationic nanoparticles. Before this experiment, we predicted that the amount of siRNA uptake promoted by fET would be lower than that by LFN lipoplexes, because condensed cationic nanoparticles/siRNA lipoplexes could show greater binding to cell surface, which may in turn facilitate cellular uptake of siRNA.

Cells were treated with fET (0.34 mA cm^–2^, 15 min) in the presence of rhodamine-labeled siRNA. Three hours after fET, the cells were lysed, and the fluorescence intensity of the lysate was measured. Data were obtained by three separate experiments, and are expressed as mean ± SD. *p<0.05 versus untreated.

### 3.3. Molecular size effect on intracellular trafficking after fET

We have reported that fET could deliver siRNA and in-stem molecular beacon (ISMB) [16] into cytoplasm.[12] The difference between siRNA/ISMB and dSC iRed was attributed to their molecular sizes. Molecular sizes of siRNA/ISMB and dSC iRed were *c*.14,000, 11,200 and 225,000, respectively. Thus, significantly larger molecular size of dSC iRed than siRNA and ISMB could prevent endosomal escape after fET-mediated endocytosis.

**Figure 4.**
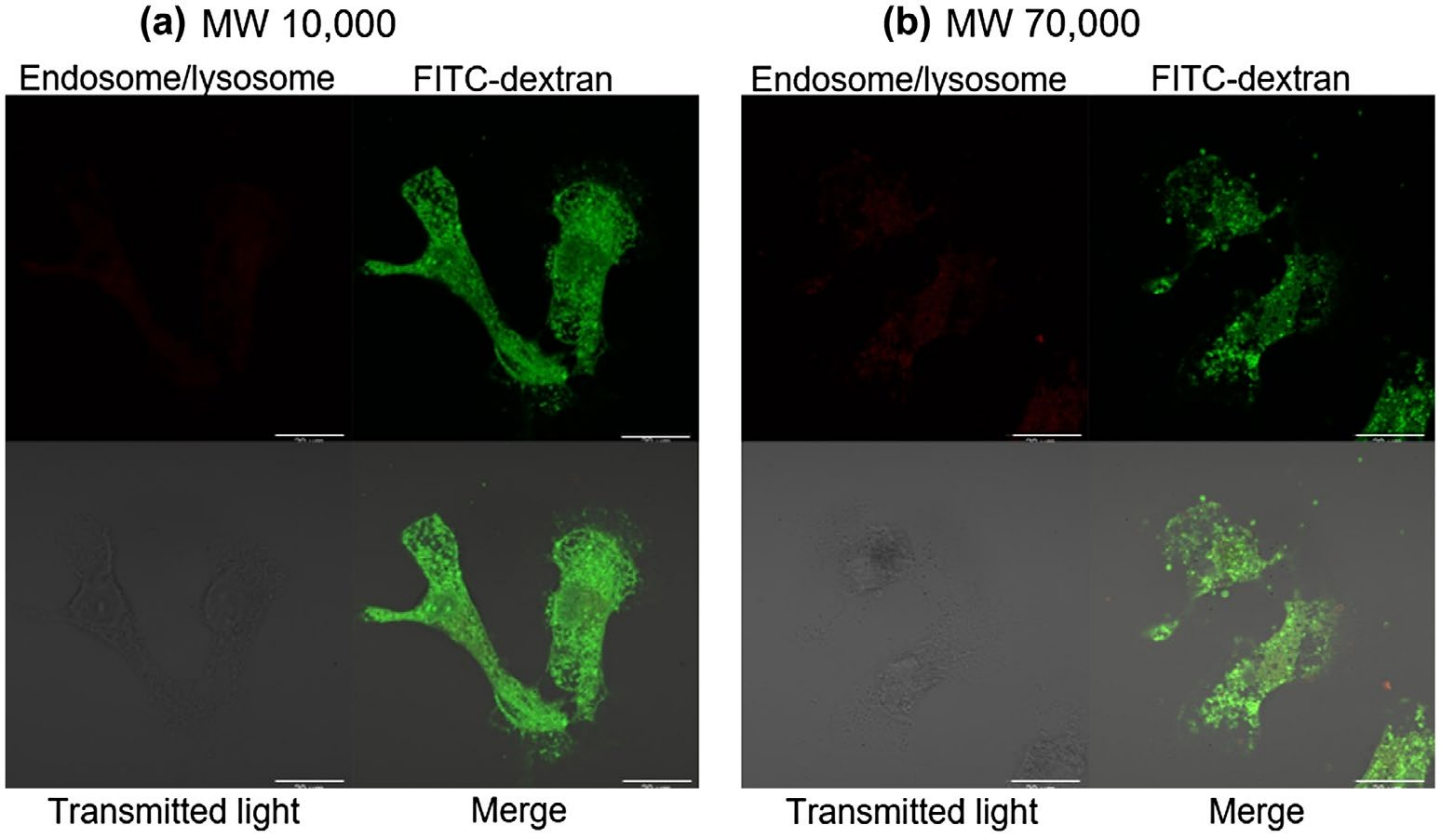
Confocal microscopy of cells 24 h after fET in the presence of (a) FITC-dextran10,000 or (b) FITC-dextran70,000.

To confirm the effect of molecular size on cytoplasmic delivery by fET, intracellular trafficking of different size molecules 24 h after fET was observed. In this experiment, FITC-labeled dextran average MW10,000 (FITC-dextran10,000) and FITC-labeled dextran average MC70,000 (FITC-dextran70,000) were used, and fET was performed. As shown in Figure 4(a), potent green signals indicating FITC-dextran10,000 were observed widely in the cells (Figure 4(a)), although red signals indicating lysosome/endosome were very faint. But, in cells without fET, red signals were clearly observed (Figure S1). These results suggest that property of endosomes changed to be leaky after fET, and FITC-dextran 10,000 was released from such leaky endosome. On the other hand, FITC-dextran70,000 was observed as many dots (Figure 4(b)), suggesting that FITC-dextran70,000 still remained in the endosome even 24 h after fET. Thus, it was suggested that endosomal escape efficiency of materials internalized by fET depends on the molecular size.

Cells were treated with fET (0.34 mA cm^–2^, 15 min) in the presence of FITC-dextran solution. After 23.5 h, the cells were stained with LysoTracker Red DND for 0.5 h, then observed with a confocal laser scanning microscope. The scale bars indicate 20 μm.

### 3.4. Enhanced transfection of dSC iRed via fET through improved endosomal escape

**Figure 5.**
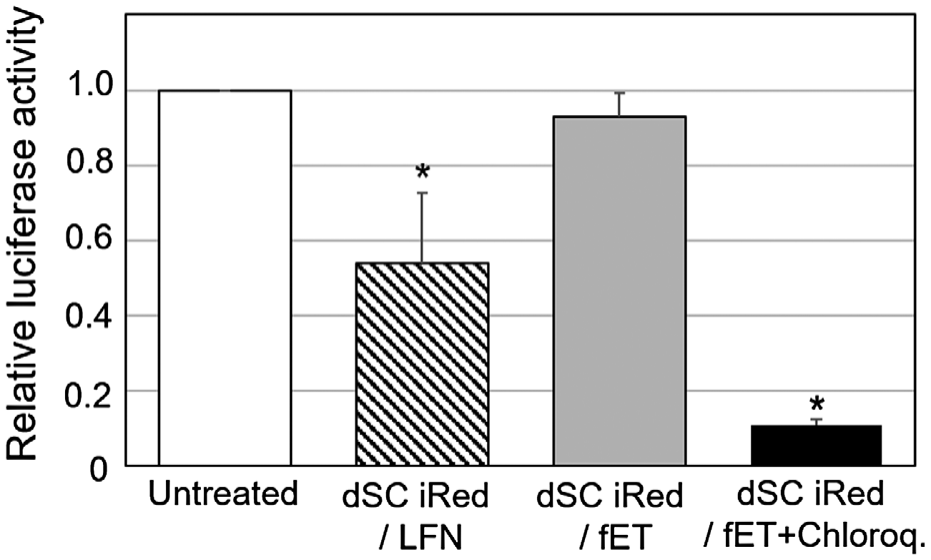
Effect of chloroquine on relative luciferase activity of the cells after transfection of dSC iRed with fET.

To improve endosomal escape of dSC iRed taken up by endocytosis, fET with dSC iRed was performed in the presence of chloroquine, which is a known lysosomotropic agent that accumulates in endosomes and lysosomes and enhances cytoplasmic delivery of various compounds.[17,18] The relative luciferase activity of the cells treated by dSC iRed/fET in the presence of chloroquine was significantly lower than that of dSC iRed/LFN lipoplexes, while dSC iRed/fET without chloroquine showed no decrease (Figure 5). In addition, the relative luciferase activity of the cells after fET with chloroquine was lower than that of the lipoplexes even in the presence of chloroquine, although the RNAi effect of dSC iRed/LFN lipoplexes was also improved by chloroquine (Figure S2). Therefore, given that the amount of extraneous macromolecules taken up following fET was significantly higher than that with the lipoplex system (Figure 3), the RNAi effect in cells exposed to fET would be expected to be higher than that of LFN-treated cells. Furthermore, dSC iRed delivered with cationic nanoparticles must be released from lipoplexes in order to exert its effects, while fET can deliver naked dSC iRed. The cationic non-viral delivery system could also interact with shRNA produced from dSC iRed to prevent an RNAi effect. Based on these results, fET would be a useful technology to deliver functional nucleic acids without compromising their activity. Thus, a combination of dSC iRed with fET in the presence of chloroquine could be applied for RNAi therapy against various diseases, such as ocular disorders and tumors.[19]

Luciferase activity of cells expressing luciferase was measured 24 h after fET (0.34 mA cm^–2^, for 15 min) in the presence of dSC iRed solution. The LFN/ dSC iRed lipoplex was used as a positive control. The concentration of chloroquine was 100 μM. Data were obtained by three separate experiments, and are expressed as mean ± SD. *p<0.05 versus untreated.

### 3.5. Effect of RNAi delivered by dSC iRed/fET on pathological adipocyte phenotype

**Figure 6.**
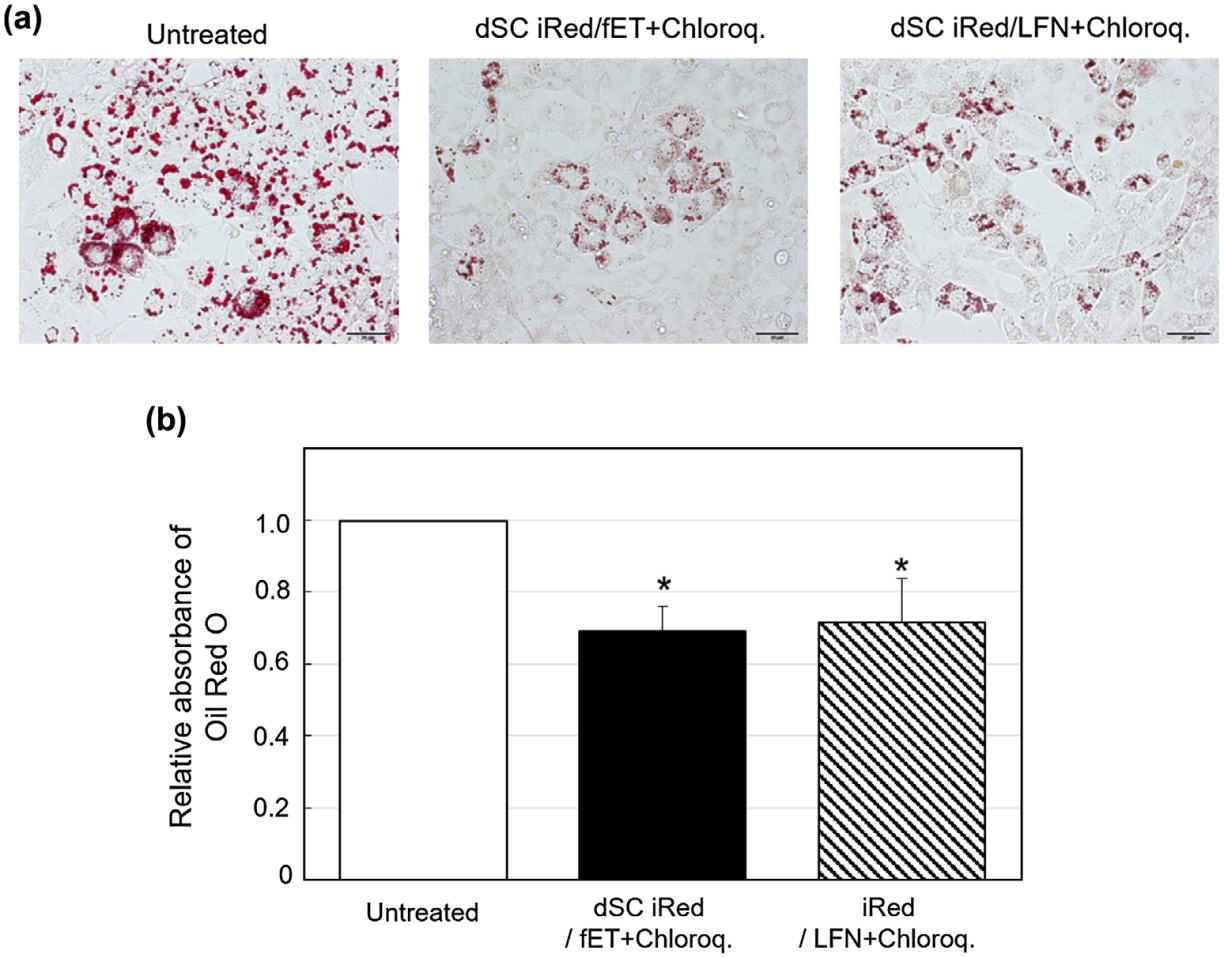
Effect on lipid accumulation in 3T3-L1 adipocytes following fET-mediated transfection of dSC iRed against resistin in the presence of chloroquine.

**Figure 7.**
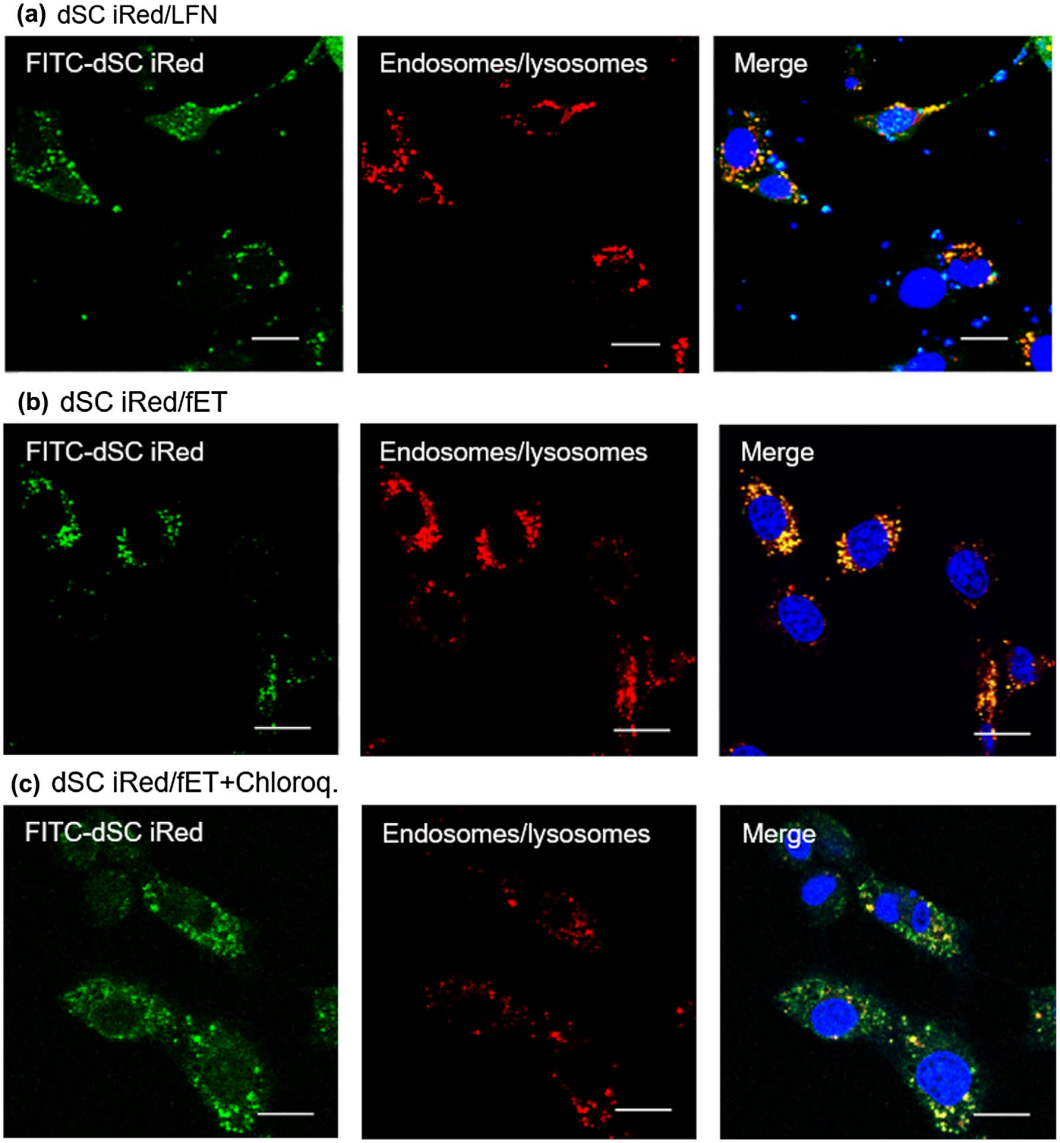
Confocal images of intracellular fate of dSC iRed.

To evaluate possible uses of dSC iRed/fET in RNAi therapy, we examined the effect of fET with iRed encoding anti-resistin shRNA on adipocyte maturation. We previously reported that some maturation processes of 3T3-L1 adipocytes involve lipid droplet accumulation in response to the key adipokine resistin [15] (Figure S3). We also showed that transfection of anti-resistin siRNA by a commercially available transfection reagent reduced lipid accumulation during adipocyte maturation.[15] Thus, RNAi targeted to the resistin gene may be useful for preventing obesity, which is one known cause of metabolic syndrome.[20] Here we induced maturation of 3T3-L1 adipocytes and then treated the cells with fET and dSC iRed against resistin. The amount of lipid droplet accumulation in the cells on day 4 after induction of maturation was significantly reduced by fET with dSC iRed in the presence of chloroquine (Figure 6(a)). Meanwhile, absorbance of Oil Red O extracted from adipocytes, which is an indicator of intracellular lipid accumulation, was suppressed 35% by dSC iRed/fET with chloroquine (Figure 6(b)). Although the suppression level of Oil Red O absorbance by fET was almost the same as that for LFN with chloroquine (Figure 6(b)), the reduction in Oil Red O staining after fET appeared to be more homogeneous than that seen for LFN (Figure 6(a)). On the other hand, no effect of dSC anti-resistin iRed/fET without chloroquine on the level of Oil Red O absorbance in 3T3-L1 cells was observed (Figure S4), although the Oil Red O absorbance was inhibited by dSC anti-resistin iRed/fET with chloroquine shown in Figure 6. Furthermore, intracellular trafficking of FITC-labeled dSC iRed in 3T3-L1 cells after fET was examined by confocal laser scanning microscopy. As shown in Figure 7, green dots (FITC-labeled dSC iRed) co-localized with red dots (endosomes/lysosomes) in the cells after fET without chloroquine (Figure 7(b)). On the other hand, the green fluorescence was widely distributed in the cells after fET with chloroquine (Figure 7(c)). These results indicate that endosomal escape of iRed was difficult without chloroquine even after fET. These results are consistent with the effect of anti-luciferase iRed on luciferase activity with/without chloroquine in B16-F1-Luc cells. Thus, the effect of molecular size on fET-mediated cytoplasmic delivery may be a common phenomenon in both B16-F1 cells and 3T3-L1 cells. These results suggest that fET of dSC iRed against resistin in the presence of chloroquine would be a useful method for regulating lipid accumulation during adipocyte maturation.

After transfection of dSC iRed against resistin by fET in the presence of chloroquine, 3T3-L1 adipocytes were fixed and stained with Oil Red O. (a) Microscopy images of cells stained with Oil Red O. Scale bars indicate 50 μm. (b) Relative absorbance of Oil Red O dye extracted from cells after Oil Red O staining. Data were obtained by three separate experiments, and are expressed as mean ± SD. *p<0.05 versus untreated.

Cells were treated with FITC-labeled dSC iRed/lipoplex (a) or fET (0.34 mA cm^-2^ for 15 min) with or without chloroquine in presence of FITC-labeled dSC iRed (b, c). After the incubation for 3 h, cells were observed by a confocal laser scanning microscope. Intracellular distribution of FITC-labeled dSC (green) and endosomes/lysosomes stained with LysoTracker Red (red) were shown as individual images, respectively. In the merged images, nuclei were stained with Hoechst 33342 (blue) to recognize intracellular positions of FITC-labeled dSC and endosome/lysosomes. Scale bars indicate 20 μm.

## 4. Conclusions

Here we examined the use of fET to facilitate cytoplasmic delivery of the novel shRNA expression system dSC iRed. Efficient reduction in expression of genes targeted by dSC iRed derived shRNA could be improved by the addition of chloroquine, which promotes endosomal escape of dSC iRed. As such, dSC iRed/fET with chloroquine showed a significantly higher RNAi effect than dSC iRed/LFN lipoplexes. Furthermore, fET of dSC iRed carrying shRNA against resistin in the presence of chloroquine suppressed lipid accumulation that occurs during adipocyte maturation. Together these results show that a combination of dSC iRed with fET is a useful method for effective regulation of target gene expression.

## Supplemental material

The supplemental material for this paper is available online at http://dx.doi.org/10.1080/14686996.2016.1221726.

## Disclosure statement

No potential conflict of interest was reported by the authors.

## Funding

This work was supported by a Grant-in-Aid for Scientific Research on Innovative Areas ‘Nanomedicine Molecular Science’ [number 2306] from the Ministry of Education, Culture, Sports, Science, and Technology of Japan.

## Supplementary Material

suppl_data.zip
