# Supplementary figures and images for: The novel functional nucleic acid iRed effectively regulates target genes following cytoplasmic delivery by faint electric treatment
Source: Sci Technol Adv Mater. 2016 Sep 16;17(1):554–62. doi: 10.1080/14686996.2016.1221726 (PMC5111564; doi:10.1080/14686996.2016.1221726)

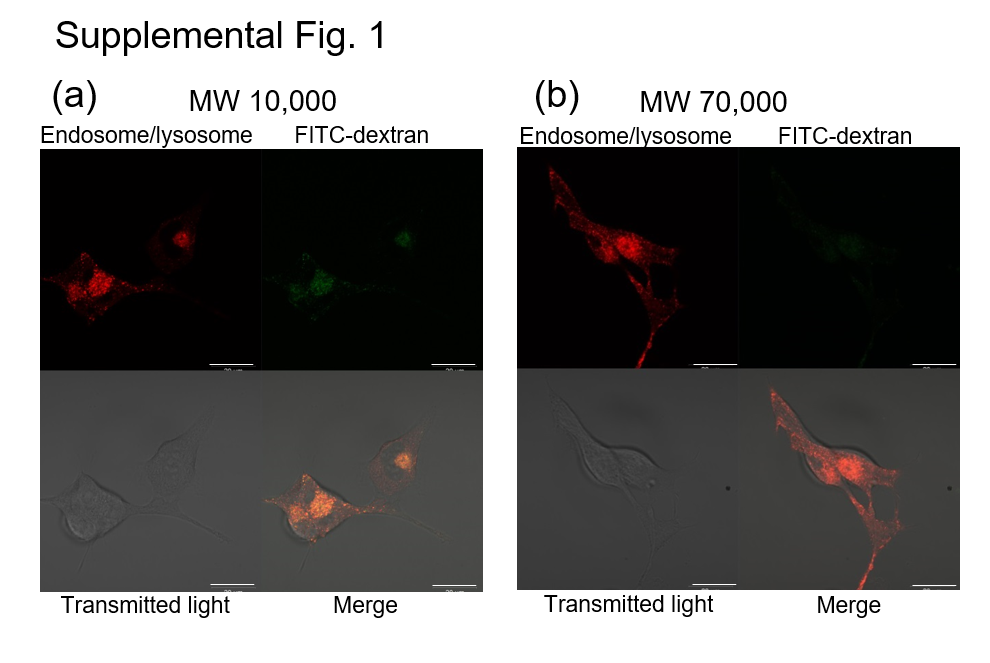

Supplement: suppl_data.zip [file tsta_a_1221726_sm2163.zip › suppl_data/supple figure1.tif]

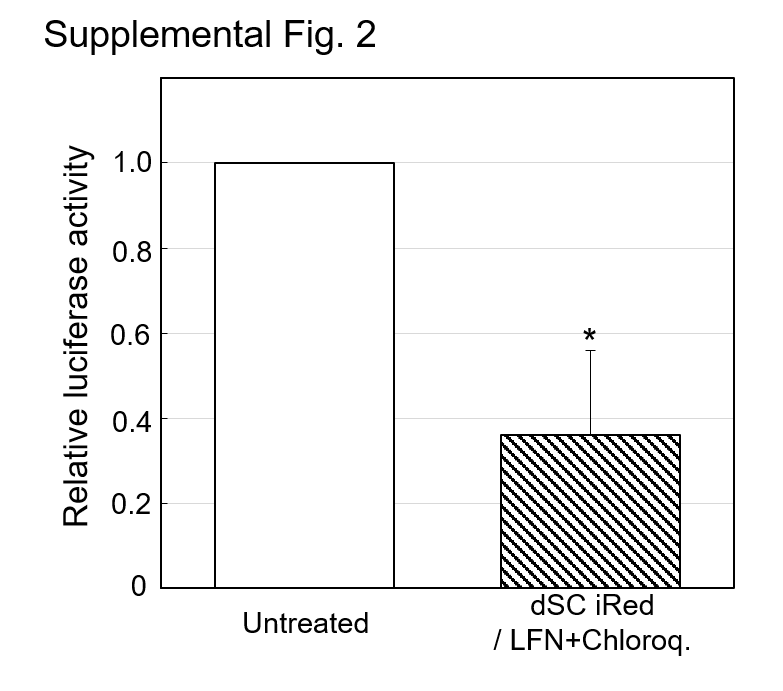

Supplement: suppl_data.zip [file tsta_a_1221726_sm2163.zip › suppl_data/supple figure2.tif]

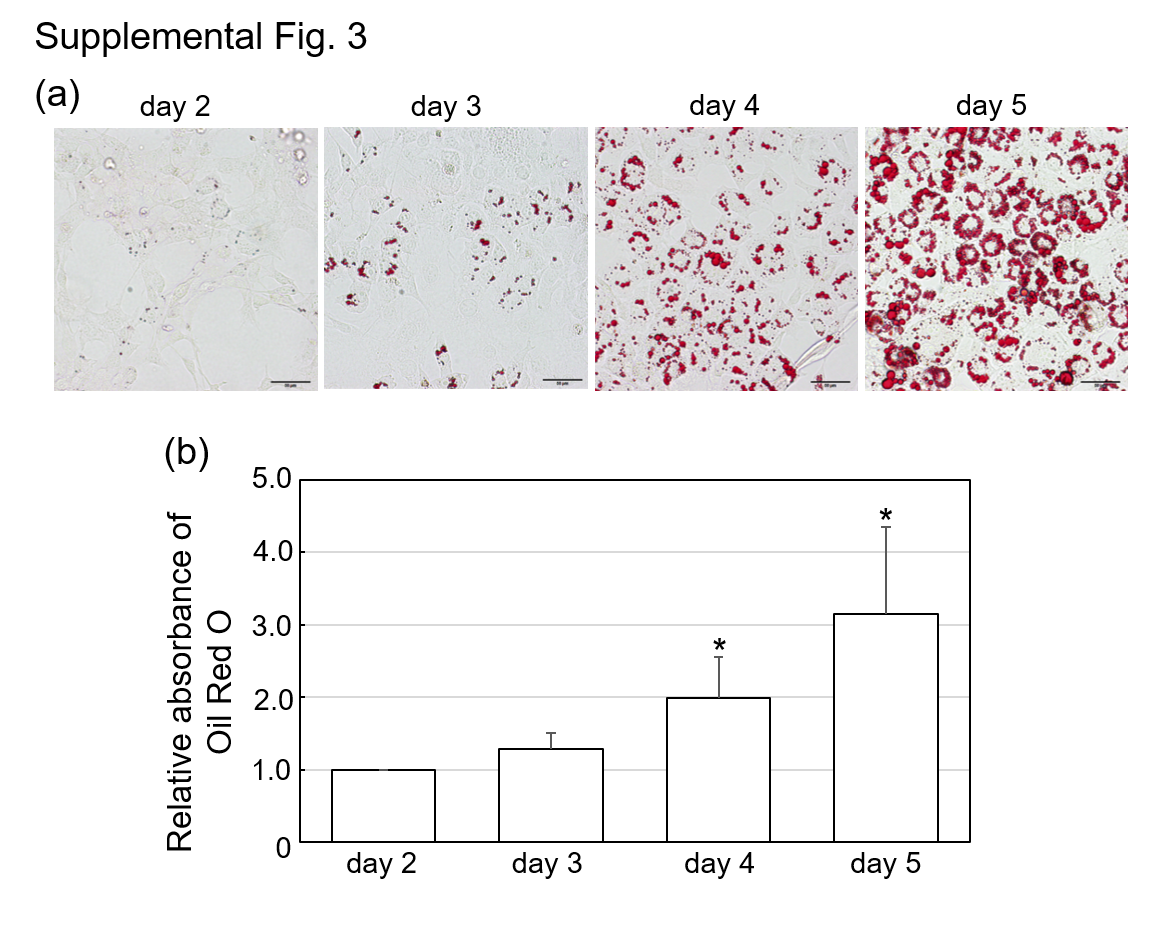

Supplement: suppl_data.zip [file tsta_a_1221726_sm2163.zip › suppl_data/supple figure3.tif]

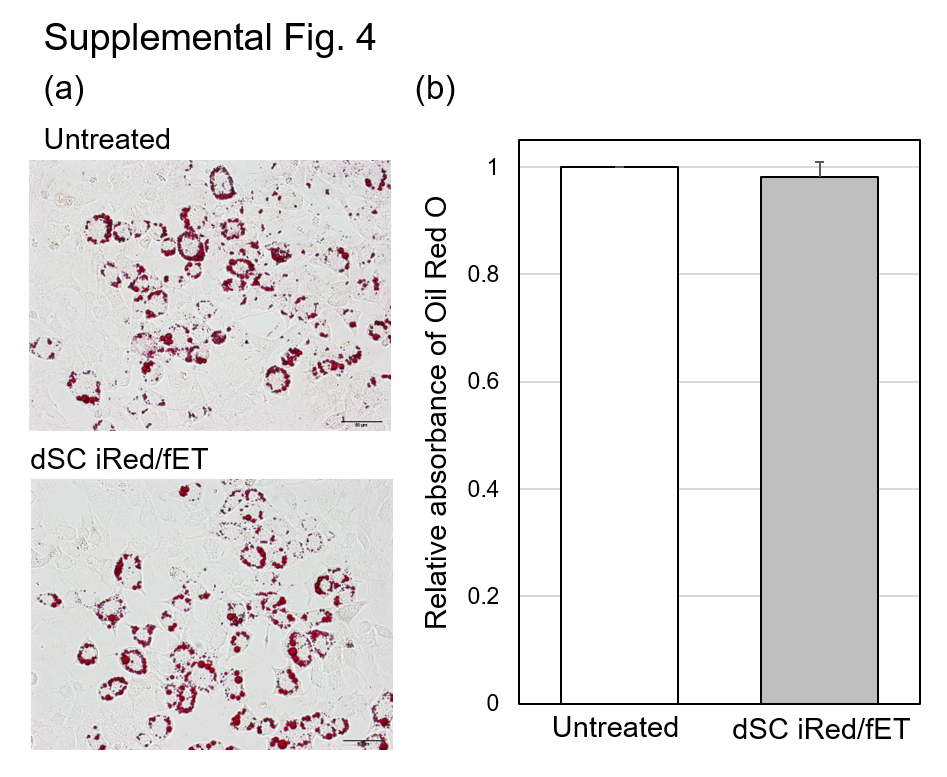

Supplement: suppl_data.zip [file tsta_a_1221726_sm2163.zip › suppl_data/supple figure4.tif]
